# Effect of school feeding program on the anthropometric and haemoglobin status of school children in Sidama region, Southern Ethiopia: a prospective study

**DOI:** 10.1017/jns.2022.73

**Published:** 2022-08-24

**Authors:** Tsion A. Desalegn, Samson Gebremedhin, Barbara J. Stoecker

**Affiliations:** 1 School of Nutrition, Food Science and Technology, Hawassa University, Hawassa, Ethiopia; 2 School of Public Health, Addis Ababa University, Addis Ababa, Ethiopia; 3 Department of Nutritional Sciences, Oklahoma State University, Stillwater, OK, USA

**Keywords:** Anthropometric status, Ethiopia, Haemoglobin, School feeding program, School meals, BAZ, BMI-for-age *z*-score, DID, Difference-in-Differences, HAZ, height-for-age *z*-score, HGSF, home-grown school feeding, SFP, School feeding program

## Abstract

Ethiopia recently scaled up the implementation of a school feeding program **(**SFP) as a targeted intervention for protecting disadvantaged school children from hunger and food insecurity. However, the contribution of the program to advancing the nutritional status of children has not been adequately explored. We assessed the effect of SFP on the anthropometric and haemoglobin status of school children in Sidama Region, Southern Ethiopia. Our prospective cohort study compared the height-for-age *z*-score (HAZ), BMI-for-age *z*-score (BAZ) and haemoglobin concentration of SFP beneficiary (*n* 240) and non-beneficiary (*n* 240) children, 10–14 years of age. The children were recruited from 8 SFP implementing and 8 control schools using a multistage sampling procedure and were followed for an academic year. The SFP intervention and control schools were matched one-to-one based on agro ecological features and geographical proximity. Exposure, outcome and pertinent extraneous variables were collected through baseline and end-line surveys. Multilevel difference-in-differences (DID) analysis was used to measure the net effect on the outcomes of interest. In the multivariable DID model adjusted for potential confounders including maternal and paternal literacy, household monthly income, wealth index and household food insecurity, the SFP did not show significant effects on the haemoglobin concentration (*β* = 0⋅251, 95 % confidence interval (CI): −0⋅238, 0⋅739), BAZ (*β* = 0⋅121, 95 % CI: −0⋅163, 0⋅405) and HAZ (*β* = −0⋅291, 95 % CI: −0⋅640, 0⋅588) of children.

## Introduction

A School Feeding Program (SFP) is a targeted social protection program for safeguarding vulnerable children from hunger and has multiple side benefits^(1–3)^. At the individual level, an SFP improves school enrolment, attendance and academic performance and may advance the anthropometric status of children^(3,4)^. At the household level, it alleviates food insecurity and economic stress^(5)^. At the community level, a home-grown school feeding (HGSF) provides market opportunities to smallholder farmers and stimulates the local economy^(6)^.

Almost all countries in the world implement school feeding at different scales and quality^(2)^. In 2013, globally 368 million children were fed daily at school with an annual investment of 47–75 billion USD^(7)^. Investment per child ranged from 20 to 1500 USD per year^(7)^. SFP has been operated in two modalities: school meals where children are fed in the school compound or take-home rations, where families are given food as incentives for their children's school enrolment. The SFP frequently can be integrated with other school health services including deworming, nutrition education and micronutrient supplementation^(7)^.

In Ethiopia, an SFP was first piloted in 1994 in Tigray region^(8)^ and the HGSF program was initiated in 2012 in the southern region^(9)^. The coverage of the program has progressively increased and in 2016 more than a million children were reached^(9)^. Currently, the government has expanded HGSF to different parts of the country including urban areas and the program is being considered as a key nutrition-sensitive intervention to combat malnutrition^(10)^.

So far, only a few cross-sectional studies have evaluated the effect of the Ethiopian SFP on the nutritional status of children^(11,12)^. In Southern Ethiopia, Zenebe *et al.* reported significantly better body-mass-index for age (BAZ) and height-for-age (HAZ) *Z*-scores among SFP beneficiaries^(11)^. A cross-sectional study in Addis Ababa also found better anthropometric and haemoglobin status among children covered by SFP^(12)^. A systematic review of SFP evaluations in low- and middle-income countries reported small but significant effect on the weight gain of children^(13)^.

The purpose of the present study was to evaluate the effect of SFP on the anthropometric and haemoglobin status of school children in southern Ethiopia where HGSF is being implemented.

## Materials and methods

### Study design

A prospective cohort study was used to compare the anthropometric and haemoglobin status of SFP beneficiary and non-beneficiary school children 10–14 years of age. SFP beneficiaries and non-beneficiaries of the program were enrolled from 16 schools and followed for one academic year. Data on exposure, outcome and other pertinent control variables were gathered over two rounds of baseline and end-line surveys. The net effect of the program on the outcomes of interest was estimated using Difference-in-Differences (DID) analysis.

### Study setting

The study was conducted in 16 second-cycle primary schools (SCPS) found in four SFP-targeted rural districts (Borecha, Dara, Bona and Loka Abaya) of Sidama region, southern Ethiopia. The region is located approximately 300 km south of the national capital, Addis Ababa, and has about four million inhabitants, of whom 95 % were rural dwellers^(14)^. The zone covers nearly 10 000 km^2^ area and is characterised by diverse agroecological features. The region is divided into 23 administrative districts.

In Ethiopia, the primary education system is divided into first- (grade 1–4) and second- (grade 5–8) cycles. During the study, 111 SCPS including 27 SFP-targeted schools were operational in the four districts. Schools are typically enrolled in the program in consideration of the severity of the food insecurity situation in their catchment areas, as evaluated by the regional education bureau, donors and other partners. Students registered in SFP-targeted schools receive a daily free cooked school meal prepared from cereals and legumes.

### Sample size calculation

The sample size of 480 school children (240 SFP beneficiaries and 240 non-beneficiaries) was determined using G*Power software^(15)^ assuming mean BAZ, HAZ and haemoglobin would be compared between the two groups using a one-tailed mean difference *t*-test at 95 % confidence level and 80 % power. Furthermore, one-to-one allocation ratio, medium effect size (*d* 0⋅4), design effect of 2 and 20 % allowance for possible dropout were assumed. The sample size calculation formulas in consideration of each objective of the study were presented later (Table 1).

**Table 1.** Sample size calculation for each objective of the study

Objectives	Calculation approach	Input for calculation	Sample size
1. Effect of the program on underweight status	Double proportion formula for double cohort study. Computed using Epi info 7 software	95 % confidence level, 80 % power, 20 % expected prevalence of underweight relative risk of 0⋅5 (Medium effect size) one-to-one allocation ratio, 20 % non-response rate.	240 SFP beneficiaries 240 controls
2. Effect of the program on haemoglobin status	Sample size formula for comparison of two independent means. Computed using g*power software	Medium effect size (*d* 0⋅4), 95 % confidence level, 80 % power, one-to-one allocation ratio, 20 % non-response rate	168 SFP beneficiaries 168 controls

### Sampling procedure

Students were identified from eight SFP-targeted and eight control schools from the above-mentioned four districts. Students registered in SFP-targeted schools were considered as SFP beneficiaries, whereas, those from non-targeted schools were taken as non-beneficiaries.

From each of the four districts, two beneficiary and two non-beneficiary schools, totally sixteen schools, were included in the study. In every district, two schools with SFP were selected at random from schools enrolled in the program. Then each of the selected schools with the feeding program was one-to-one matched with a control school based on predefined matching criteria: similar agro-ecology features (similar in agricultural production, climatic zone and landscape) and geographical proximity (being within the same district). Ultimately 480 children, 30 eligible students per school, were selected across all the grades using a simple random sampling technique. Purposely to increase the sample size, those who were not willing to take part in the study were replaced by randomly selected eligible children from the same section.

### Variables of the study

The exposure variable of interest was SFP enrolment status (beneficiary *v.* non-beneficiary) and the outcome variables were BAZ, HAZ and haemoglobin concentrations of the children. Control variables were socio-economic status indicators including maternal and paternal literacy, household monthly income, household wealth index, household food insecurity, head of the household (male *v*. female), type of water source at home (improved *v*. non-improved), age and sex of the child.

### Methods of data collection

Data were collected in two rounds of surveys implemented at the beginning (September 2017) and end (June 2018) of the 2017/2018 academic year. A similar set of variables were collected in the surveys. Haemoglobin status was measured in the field from capillary blood using HemoCue analyzer (HemoCue Hb 301, Ängelholm, Sweden). Haemoglobin measurements were adjusted for altitude according to the recommendation of the World Health Organization (WHO)^(16)^. Height and weight measurements were taken following standard procedures without heavy clothing and shoes using a portable stadiometer and calibrated digital UNICEF scale. Haemoglobin and anthropometric measurements of children were taken in private settings in each school.

Parents of the index children were interviewed at home about socio-demographic, economic, access to social services and household food security situations using a structured and pretested questionnaire prepared in the local *Sidamu Afoo* language. The parts of the questionnaires on socio-demographic and economic information were adopted from the standard Demographic Health Survey (DHS) questionnaire^(17)^. Household food insecurity was measured in accordance with the Food and Nutrition Technical Assistance (FANTA) indicator guide and classified as food secure and mild, moderate or severe food insecurity^(18)^. In accordance with the criteria of WHO-UNICEF, type of drinking water source was categorised as improved or unimproved^(19)^.

### Data analysis

SPSS version 23 software was used to analyse the data. Anthropometric indices were generated using WHO AnthroPlus software based on WHO-2007 population growth reference data. Adolescent children with *z*-score below −2 for BMI-for-age and height-for-age indices were considered as thin and stunted, respectively. As commonly done in national Demographic and Health Surveys^(20)^, household wealth index – a proxy indicator of household cumulative living standard – was developed using Principal Component Analysis (PCA) based on ownership of durable household assets and materials used for household construction.

DID analysis was used to estimate the net effect of SFP on the three outcomes of interest. DID is an analytic technique for estimating the effect of an intervention based on observational data by taking into consideration the baseline difference between the intervention and control groups and changes overtime in the groups^(21)^. In the present analysis, DID was estimated using simple and multiple mixed-effects linear regression model with a random intercept defined for each school. Initially, the balance of the control variables between the intervention and control groups was assessed using *χ*^2^ and independent sample *t*-tests and in the multivariable models, significantly unbalanced variables were adjusted.

### Ethical considerations

The study was implemented in accordance with the Declaration of Helsinki for research involving human subjects. The study protocol was approved by Hawassa University Ethics Committee (Reference No; IRB/003/10) and data were collected after taking written informed consent from the parents of the children. Assent was also secured from the children themselves.

## Results

### Socio-demographic characteristics

Table 2 summarises the characteristics of the 240 SFP beneficiaries and 240 non-beneficiary children and their caregivers. The mean (±sd) age of the caregivers during the baseline survey was 32⋅8 (±9⋅3) and 32⋅1 (±8⋅2) years among SFP beneficiary and non-beneficiary children, and the difference was not significant (*P* = 0⋅905). The two groups were comparable based on age and sex; however, caregivers of the non-beneficiary children had significantly better educational and economic status and also had better access to improved drinking water sources. The two groups were also significantly different based on religion, marital status, household food insecurity and monthly income (Table 2).

**Table 2.** Basic characteristics of school feeding program beneficiary and non-beneficiary children and their caregivers, Sidama region, southern Ethiopia, 2017

Socio-demographic characteristics	SFP beneficiaries (*n* 240)		SFP non-beneficiaries (*n* 240)		*P*-value
	Freq	%	Freq	%	
Age of the caregiver (years)					
15–24	44	18⋅3	41	17⋅1	0⋅905
25–34	88	36⋅7	92	38⋅3	
35 or above	108	45⋅0	107	44⋅6	
Maternal education					
No formal education	125	52⋅1	127	52⋅9	<0⋅001^a^
Primary education	33	13⋅8	6	2⋅5	
Secondary or above	82	34⋅2	107	44⋅6	
Paternal education					
No formal education	122	50⋅8	108	45⋅0	<0⋅001^a^
Primary education	58	24⋅2	20	8⋅3	
Secondary or above	60	25⋅0	112	46⋅7	
Marital status of the caregiver					
Married	216	90⋅0	231	96⋅3	0⋅007^a^
Others	24	10⋅0	9	3⋅8	
Household wealth index					
Poor	74	30⋅8	61	25⋅4	0⋅005^a^
Medium	110	45⋅8	90	37⋅5	
Rich	56	23⋅3	89	37⋅1	
Household food insecurity					
Secure	50	20⋅8	53	22⋅1	0⋅019^a^
Mild	15	6⋅3	35	14⋅6	
Moderate	167	69⋅6	143	59⋅6	
Severe	8	3⋅3	9	3⋅8	
Religion					
Protestant	220	91⋅7	189	78⋅8	<0⋅001^a^
Others	20	8⋅3	51	21⋅3	
Average monthly income (ETB)					
Less than 1000	156	65⋅0	121	50⋅4	<0⋅001^a^
1000–2000	69	28⋅8	115	47⋅9	
2000 or above	15	6⋅3	4	1⋅7	
Water source					
Non-improved	233	97⋅1	205	85⋅4	<0⋅001^a^
Improved	7	2⋅9	35	14⋅6	
Sex of the child					
Boy	132	55⋅0	123	51⋅3	0⋅410
Girl	108	45⋅0	117	48⋅8	
Age of the child (years)					
10	40	16⋅7	46	19⋅2	0⋅929
11	54	22⋅5	53	22⋅1	
12	52	21⋅7	47	19⋅6	
13	46	19⋅2	49	20⋅4	
14	48	20⋅0	45	18⋅8	

ETB – Ethiopian birr, at the time of the study 1 USD was roughly equivalent to 30 ETB.

a Significant difference at *P*-value of <0⋅05 (*χ*^2^ test).

Among 480 students enrolled in the study, 17 (3⋅5 %) dropped out of the study; accordingly, they had no measurements for the outcome variables. Dropout rate was significantly lower among SFP beneficiaries (1⋅3 %) as compared to non-beneficiaries (5⋅8 %) (*P* = 0⋅007).

### Effect of SFP on haemoglobin and anthropometric status of school children

Table 3 compares the haemoglobin and anthropometric status of SFP beneficiary and non-beneficiary children during the baseline and end line surveys. At baseline, SFP beneficiaries had significantly lower haemoglobin level (*P* < 0⋅001) but higher height (*P* = 0⋅012) and HAZ score (*P* = 0⋅03). During the end-line survey, the mean haemoglobin in SFP beneficiaries remained significantly lower (*P* < 0⋅001) but there were no statistically significant differences in terms of the other parameters (Table 3). However, in the sex-stratified analysis, SFP beneficiary males have significantly different BAZ values at end-line survey (*P* = 0⋅001) (Supplementary Table S1).

**Table 3.** Anthropometric and haemoglobin status of SFP beneficiary and non-beneficiary children during baseline and end line surveys, Sidama region, southern Ethiopia, 2017

Parameters	Baseline survey			End line survey		
	SFP beneficiaries (*n* 240)	Non-beneficiaries (*n* 240)	*P*-value^b^	SFP beneficiaries (*n* 237)	Non-beneficiaries (*n* 226)	*P*-value^b^
	Mean (±sd)	Mean (±sd)		Mean (±sd)	Mean (±sd)	
Hgb (g/dl)	12⋅4 (±2⋅1)	13⋅9 (±1⋅7)	<0⋅001^a^	12⋅7 (±2⋅1)	13⋅9 (±1⋅6)	<0⋅001^a^
Height (cm)	146⋅1 (±9⋅0)	144⋅2 (±7⋅6)	0⋅012^a^	146⋅5 (±8⋅4)	146⋅3 (±8⋅2)	0⋅816
Weight (kg)	34⋅6 (±6⋅6)	33⋅7 (±6⋅4)	0⋅128	35⋅4 (±6⋅1)	35⋅3 (±6⋅2)	0⋅956
HAZ	−0⋅53 (±1⋅51)	−0⋅79 (±1⋅48)	0⋅03^a^	−0⋅74 (±1⋅42)	−0⋅74 (±1⋅59)	0⋅628
BAZ	−1⋅06 (±1⋅20)	−0⋅98 (±1⋅22)	0⋅351	−0⋅95 (±1⋅1)	−0⋅88 (±1⋅28)	0⋅539
Stunting (%)	22⋅1	23⋅8	0⋅644	21⋅1	20⋅4	0⋅844
Thinness (%)	22⋅2	20⋅4	0⋅735	14⋅3	19⋅5	0⋅176

a Statistically significant difference at <0⋅05 level of significance.

b *t* or *x*^2^ test.

The bi variable and multivariable DiD analyses did not show a significant effect of the SFP, neither on the haemoglobin nor anthropometric status of school children. In the multivariable model adjusted for potential confounders including maternal and paternal literacy, household monthly income, wealth index, household food insecurity, type of drinking water source and religion, SFP did not show a significant net effect on haemoglobin status (*β* = 0⋅251, 95 % confidence interval (CI): –0⋅238, 0⋅739), BAZ (*β* = 0⋅121, 95 % CI: –0⋅163, 0⋅405) and HAZ (*β* = –0⋅291, 95 % CI: –0⋅640, 0⋅588) (Table 4)

**Table 4.** Effect of a school feeding program on haemoglobin and anthropometric status of school children, Sidama region, Southern Ethiopia, 2017

Parameters	Crude		Adjusted*	
	*β* (95 % CI)	*P*-value	*β* (95 % CI)	*P*-value
Haemoglobin (g/dl)	0⋅253 (−0⋅242, 0⋅747)	0⋅317	0⋅251 (−0⋅238, 0⋅739)	0⋅314
BAZ	−0⋅103 (−0⋅164, 0⋅406)	0⋅405	0⋅121 (−0⋅163, 0⋅405)	0⋅404
HAZ	−0⋅291 (−0⋅648, 0⋅664)	0⋅111	−0⋅291 (−0⋅640, 0⋅588)	0⋅103

* Adjusted for maternal and paternal literacy, household monthly income, wealth index, household food insecurity, type of drinking water source and religion of the caregiver.

## Discussion

The overarching goal of school feeding programs in low- and middle-income countries is the protection of vulnerable children from hunger and food insecurity. SFP may also have other side benefits including improving the nutritional status of children^(13,22–24)^. Nevertheless, in this prospective cohort study, we observed no statistically significant effects of the program on the anthropometric and haemoglobin status of school children.

We found that children who received school meals did not gain weight or height significantly better than their counterparts. This is counter to the conclusion of a systematic review that SFP in low- and middle-income countries has a small but significant effect on the weight gain of children^(13)^. Prospective observational studies or controlled trials undertaken in Bangladesh^(22)^, Jamaica^(23,24)^, Kenya^(25)^ and Lao^(26)^ also confirmed that school meals significantly improve children's anthropometric status. Here it is important to note that the effectiveness of SFP implemented in different settings cannot be directly compared to each other due to remarkable differences in investment and implementation quality^(7)^. During the field work, the researcher observed multiple problems with the implementation of the school feeding programs in the study area. The school feeding programs did not start at the beginning of the academic year. The duration of the intervention period was 7 months (from December to the end of June). All schools had a problem with water supply, and sometimes because of water shortages; the meal of the day was skipped. In addition, in some schools, students shared food because of shortage of dishes and all these factors might affect the quality of the SFP.

Based on survey data, two studies in Ethiopia have reported positive effects of SFP on the anthropometric status of children^(11,27)^. A comparative cross-sectional study in southern Ethiopia found improved dietary diversity, BAZ and HAZ scores among children who received meals at school than their counterparts^(11)^. Likewise, in northern Ethiopia, children not enrolled in the feeding program had two times increased risk of thinness but there was no meaningful difference in the risk of stunting^(12,27)^. However, as both studies used cross-sectional design, they are likely to be affected by systematic errors including confounding and selection bias.

We also observed that SFP had no significant effects on the haemoglobin level of school children. So far, only a few studies have explored the effect of SFP on the haemoglobin level or anaemia status of children. A one-year prospective cohort study in an urban slum in Kenya found that children participating in the SFP had lower levels of anaemia that their controls^(28)^. In a cluster randomised controlled trial in Uganda, SFP reduced the prevalence of anaemia among adolescent girls^(29)^. In Ghana, SFP participants had a significantly higher haemoglobin concentration and the prevalence of anaemia was lowered by 10 percentage points^(30)^. In Addis Ababa, the prevalence of anaemia was reduced by 17 percentage points among SFP beneficiaries as compared to 3 percentage points among children in the control group^(12)^.

The null association observed between SFP enrolment and anthropometric and haemoglobin status of children can be explained by multiple reasons. Firstly, we followed the children for only one academic year, which may not be adequate to assess the long-term effects of the program. Secondly, the quality of school meals provided to children may not be optimal to advance the nutritional status of children In the study districts, SFP beneficiary children were frequently provided with monotonous meals prepared from cereals and legumes and access to nutrient-rich fruits, vegetables and animal source foods through the program was extremely limited and each school child in the study area received a daily hot meal prepared from 150 g of dry cereals and beans in different forms. A local food called ‘Nifro’ is a boiled mixture of maize, beans, vegetable oil and iodised salt. The other meal is called ‘Kinche’, and it is made from cracked wheat with added vegetable oil and iodised salt.

Other studies show that students who participate in school meal programs consume more whole grains, milk, fruits and vegetables during meal times and have better overall diet quality and nutritional status than nonparticipants^(31)^. In our study area, most primary schools have approximately 2–6 hectares of farmland and it is sufficient to implement school gardening. Also, money in HGSF budgets could be allocated to purchase vegetables and fruits from local farmers. Thus, the deficiencies in some key micronutrients might be addressed through linking HGSF with communities and schools to obtain micronutrient-rich local foods such as nutritious leafy fresh vegetables and fruits that are outstanding sources of vitamins and minerals.

Finally, due to logistic constraints, the initiation of the SFP was delayed for about 3 months and intermittent interruptions were also encountered during the course of the study. A previous study from southern Ethiopia has also witnessed that the SFP in the area is challenged by interruptions due to financial and logistic constraints^(11)^.

The full costs of on-site meal programs have been by collecting data from SFP implementers at all levels in four countries in Sub-Saharan Africa. As reported in 2009, costs ranged from US$28 to US$63 per child per year (weighted average US$40 per child per year)^(1)^. In Ethiopia, a HG-SFP cost analysis draft report from Partnership for Child-development in 2015 revealed that the total cost per child per year of HGSF in the SNNPR was US$ 28⋅01^(32)^. The present cost, however, might be higher due to inflation.

The following limitations need to be considered while interpreting the findings of the study. Because the study was not a randomised control trial, it was not possible to standardise the intervention including the quality of meal provided to children. Further, we were not able to exclude systematic baseline differences between the two groups. Though key socio-demographic differences between the two groups were statistically adjusted, residual confounding cannot be excluded. As reported in the results section, dropout rates were significantly lower among SFP beneficiaries than in non-beneficiaries and this may underestimate the benefit of the SFP. Finally, due to sample size concerns, we did not measure the effect of SFP separately among undernourished children.

## Conclusion

This prospective cohort study did not observe significant contributions of the SFP, as implemented in these four districts in Sidama region. To improve the anthropometric and haemoglobin status of school children SFP should provide healthy balanced meals.

## Supplementary material

For supplementary material accompanying this paper visit https://doi.org/10.1017/jns.2022.73. click here to view supplementary material

## References

[1] Bundy D, Burbano C, Grosh M, (2009) Rethinking School Feeding: Social Safety Nets, Child Development and the Education Sector. Washington, DC: The World Bank; available at https://docs.wfp.org/api/documents/WFP-0000020650/download/.

[2] Jomaa LH, McDonnell E & Probart C (2011) School feeding programs in developing countries: impacts on children's health and educational outcomes. Nutr Rev 69, 83–98. 1–9. doi:10.1111/j.1753-4887.2010.00369.x. 21294742

[3] Wang D & Fawazi WW (2020) Impacts of school feeding on educational and health outcomes of school-age children and adolescents in low- and middle-income countries: protocol for a systematic review and meta-analysis. Syst Rev 9, 55. doi:10.1186/s13643-020-01317-6. 32178734 PMC7075040

[4] Kristjansson B, Petticrew M, MacDonald B, (2007) School feeding for improving the physical and psychosocial health of disadvantaged students. Cochrane Database Syst Rev, 102. doi: 10.1002/14651858.CD004676.pub2. 17253518

[5] Lawson TM (2012) Impact of School Feeding Programs on Educational, Nutritional, and Agricultural Development Goals: A Systematic Review of Literature; available at https://www.semanticscholar.org/paper/Impact-of-School-Feeding-Programs-on-Educational%2C-A-Lawson/be537bf3d9e33a85e487528a4fa00f1618c892c8.

[6] World Food Program, Food and Agriculture Organization of the UN (2018) Home-Grown School Feeding Resource Framework Synopsis. Rome: FAO and WFP.

[7] WFP (2013) State of School Feeding Worldwide. Rome, Italy: WFP.

[8] Poppe R, Frölich M & Haile G (2019) School meals and educational outcomes in rural Ethiopia. J Dev Stud 55, 1741–1756. doi:10.1080/00220388.2017.1311405.

[9] UNICEF (2016) Ethiopia Humanitarian Situation Report #4; available at http://www.unicef.org/ethiopia/UNICEF_Ethiopia_CO_Humanitarian_Sitrep_April_2016.pdf (accessed 1 July 2019).

[10] The Federal Democratic Republic of Ethiopia (2016) National School Health and Nutrition Strategy. Addis Ababa: Ministry of Education.

[11] Zenebe M, Gebremedhin S, Henry CJ, (2018) School feeding program has resulted in improved dietary diversity, nutritional status and class attendance of school children. Ital J Pediatr 44, 16. doi:10.1186/s13052-018-0449-1. 29361948 PMC5782386

[12] Gutama M (2017-06) Nutritional Status and School Performance of Children Benefited from School Feeding Program in Selected Elementary School, Arada Sub City, Addis Ababa, Ethiopia; available at http://213.55.95.56/handle/123456789/1447.

[13] Watkins K, Gelli A, Hamdani S, 2015) Sensitive to Nutrition? A Literature Review of School Feeding Effects in the Child Development Lifecycle. Working Paper Series.

[14] Population Census Commission [Ethiopia] (2008) Summary and Statistical Report of the 2007 Population and Housing Census: Population Size by Age and Sex. Addis Ababa: Population Census Commission.

[15] Faul F, Erdfelder E, Lang AG, (2007) G*power 3: a flexible statistical power analysis program for the social, behavioral, and biomedical sciences. Behav Res Methods 39, 175–191. doi:10.3758/BF03193146. 17695343

[16] The DHS Program (2016) DHS Model Questionnaires; available at https://dhsprogram.com/Methodology/Survey-Types/DHS-Questionnaires.cfm#CP_JUMP_16175.

[17] World Health Organization. Hemoglobin Concentrations for the Diagnosis of Anemia and Assessment of Severity. vitamin and Mineral Nutrition Information System.Geneva, World Health Organization, 2011 (WHO/NMH/NHD/MNM/11.1) (http://www.who.int/vmnis/indicators/haemoglobin.pdf, accessed date January 2022).

[18] Deitchler M, Ballard T, Swindale A, (2011) Introducing a Simple Measure of Household Hunger for Cross-Cultural Use. Washington, DC: Food and Nutrition Technical Assistance II Project, AED.

[19] World Heath Origination (2017) Drinking Water: The Drinking Water Ladder. https://www.who.int/water_sanitation_health/monitoring/water.pdf.

[20] The DHS Program (2015) Wealth Index Construction. https://dhsprogram.com/programming/wealth%20index/Steps_to_constructing_the_new_DHS_Wealth_Index.pdf.

[21] Wing C, Simon K & Bello-Gomez RA (2018) Designing difference in difference studies: best practices for public health policy research. Annu Rev Public Health 39, 453–469. doi:10.1146/annurev-publhealth-040617-013507. 29328877

[22] Ahmed AU (2004) The Impact of Feeding Children in School: Evidence From Bangladesh. Washington, DC: IFPRI.

[23] Grantham-McGregor SM, Change S & Walker SP (1998) Evaluation of school feeding programs: some Jamaican examples. Am J Clin Nutr 67, 785S–789S. doi:10.1093/ajcn/67.4.785S. 9537629

[24] Powell CA, Walker SP, Chang SM, (1998) Nutrition and education: a randomized trial of the effects of breakfast in rural primary school children. Am J Clin Nutr 68, 873–879. doi:10.1093/ajcn/68.4.873. 9771865

[25] Mwaniki EW & Makokha AN (2013) Nutrition status and associated factors among children in public primary schools in Dagoretti, Nairobi, Kenya. Afr Health Sci 13, 39–46. doi:10.4314/ahs.v13i1.6. 23658566 PMC3645091

[26] Buttenheim A, Alderman H & Friedman J (2011) Impact evaluation of school feeding programmes in Lao People's Democratic Republic. J Dev Effect 3, 520–542. doi:10.1080/19439342.2011.634511.

[27] Demilew YM & Nigussie AA (2020) The relationship between school meals with thinness and stunting among primary school students, in Meket Wereda, Ethiopia: comparing schools with feeding and non-feeding program. BMC Nutr 6, 34. doi:10.1186/s40795-020-00358-3. 32793377 PMC7418431

[28] Neervoort F, Rosenstiel IV, Bongers K, (2013) Effect of a school feeding programme on nutritional status and anaemia in an urban slum: a preliminary evaluation in Kenya. J Trop Pediatr 59, 165–174. doi:10.1093/tropej/fms070. 23243080

[29] Adelman SW, Gilligan DO, Konde-Lule J, (2019) School feeding reduces anemia prevalence in adolescent girls and other vulnerable household members in a cluster randomized controlled trial in Uganda. J Nutr 149, 659–666. doi:10.1093/jn/nxy305. 30926996 PMC6461720

[30] Abizari AR, Buxton C, Kwara L, (2014) School feeding contributes to micronutrient adequacy of Ghanaian schoolchildren. Br J Nutr 112, 1019–1033. doi:10.1017/S0007114514001585. 24990068

[31] Kinderknecht K, Harris C & Jones-Smith J (2020) Association of the healthy, hunger-free kids act with dietary quality among children in the US national school lunch program. JAMA 324, 359–368. 32721008 10.1001/jama.2020.9517 PMC7388023

[32] Partnership for Child Development (PCD) (2015) Costing Analysis of School Health and Nutrition Interventions. Working paper #4; available at http://shn.cloudapp.net/Shared%20Documents/Costing%20Analysis%20of%20Enhanced%20School%20health%20Programme.pdf.

