# Supplementary material for: Effect of school feeding program on the anthropometric and haemoglobin status of school children in Sidama region, Southern Ethiopia: a prospective study
Source: J Nutr Sci. 2022 Aug 24;11:e69. doi: 10.1017/jns.2022.73 (PMC9428659; doi:10.1017/jns.2022.73)
Supplement: Supplementary file 1 [file S2048679022000738sup001.docx]

|  | **Male** | **Female** |
| --- | --- | --- |

| **Parameters** | **Baseline survey** | | | **End line survey** | | | **Baseline survey** | | | **End line survey** | | |
| --- | --- | --- | --- | --- | --- | --- | --- | --- | --- | --- | --- | --- |
|  | **SFP beneficiaries**  **(n=132)** | **Non-beneficiaries**  **(n=123)** | **P- value^†^** | **SFP beneficiaries**  **(n=131)** | **Non-beneficiaries**  **(n=113)** | **P-value^†^** | **SFP beneficiaries**  **(n=108)** | **Non-beneficiaries**  **(n=117)** | **P- value^†^** | **SFP beneficiaries**  **(n=106)** | **Non-beneficiaries**  **(n=113)** | **P-value^†^** |
|  |  |  |  |  |  |  | Mean (± SD) | Mean (± SD) |  | Mean (± SD) | Mean (± SD) |  |
|  | Mean (± SD) | Mean (± SD) |  | Mean (± SD) | Mean (± SD) |  |  |  |  |  |  |  |
| Hgb (g/dl) | 12.3(± 2.2) | 13.8(± 1.7) | <0.001^*^ | 12.7(± 2.2) | 13.86(± 1.5) | <0.001^*^ | 12.4 (± 1.9) | 13.8(± 1.6) | <0001^*^ | 12.6(± 2.0) | 14.0(± 1.7) | <0.001^*^ |
| Height (cm) | 145.1(± 8.7) | 143.1 (± 7.9) | 0.05 | 145.6(±8.4) | 145.5((± 8.7) | 0.88 | 147.3((± 8.6) | 145.3(± 7.8) | 0.06 | 147.6(± 8.4) | 147.2(±7.7) | 0.72 |
| Weight (kg) | 33.9 (± 6.1) | 34.2(±6.1) | 0.67 | 34.5(± 5.8) | 35.8(± 6.3) | 0.11 | 35.8 (± 6.5) | 33.1(± 5.1) | 0.001^*^ | 36.3(± 6.2) | 34.9 (± 5.6) | 0.09 |
| HAZ | -0.54 (± 1.5) | -0.8 (± 1.4) | 0.13 | -0.72(± 1.4) | -0.34 (± 1.7) | 0.07 | -0.48(± 1.4) | 0.75(± 1.1) | 0.11 | -070(± 1.3) | -0.98 (± 1.3) | 0.08 |
| BAZ | -1.04(± 1.1) | -0.71(± 1.2) | 0.03 | -0.97(± (1.02) | -0.51(± 1.1) | 0.001 | -0.94(± 1.1) | -1.18(± 1.0) | 0.12 | -0.91((± 1.1) | -1.19(± 1.1) | 0.06 |
| Stunting (%) | 33(25%) | 39(31.7%) | 0.23 | 29(± 22.1%) | 31(± 27.4) | 0.34 | 20 (± 18.5%) | 18(15.4%) | 0.53 | 21(19.8%) | 15(13.3%) | 0.19 |
| Thinness (%) | 30(22.2%) | 22(17.9%) | 0.33 | 22(± 16.8%) | 13(± 11.5%) | 0.24 | 21(19.4%) | 26(22.2%) | 0.35 | 12(11.3%) | 30(26.5%) | 0.04 |

Supplementary Table S1: Anthropometric data presented separately for boys and girls, Sidama region, southern Ethiopia, 2017.
